# *QuickStats:* Percentage* of Children and Adolescents Aged 5–17 Years Who Took Medication for Their Mental Health or Received Counseling or Therapy from a Mental Health Professional During the Past 12 Months,^†^ by Year — National Health Interview Survey,^§^ United States, 2019 and 2022

**DOI:** 10.15585/mmwr.mm7243a5

**Published:** 2023-10-27

**Figure:**
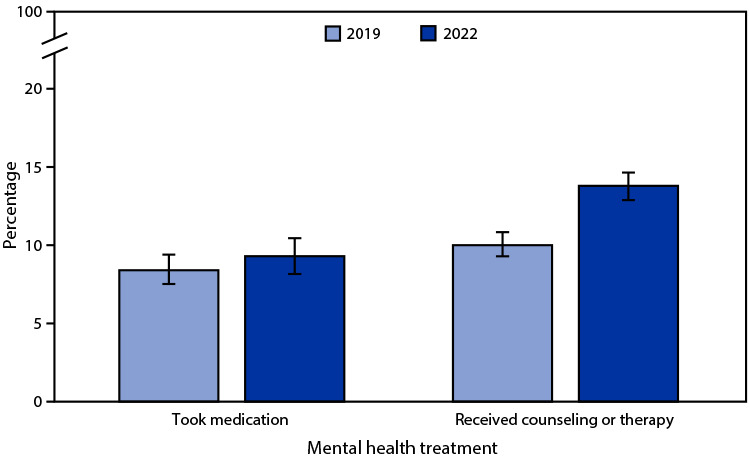
The percentage of children and adolescents aged 5–17 years who took medication for their mental health during the past 12 months did not change significantly from 2019 (8.4%) to 2022 (9.3%). The percentage of children and adolescents who received counseling or therapy during the past 12 months increased from 10.0% in 2019 to 13.8% in 2022. In both 2019 and 2022, the percentage of children and adolescents who received counseling or therapy was higher than the percentage of those who took medication for their mental health.

**Source:** National Center for Health Statistics, National Health Interview Survey, 2019 and 2022. https://www.cdc.gov/nchs/nhis/index.htm

